# Supplementary material for: Optimization of S-Nitrosocaptopril Monohydrate Storage Conditions Based on Response Surface Method
Source: Molecules. 2021 Dec 13;26(24):7533. doi: 10.3390/molecules26247533 (PMC8706474; doi:10.3390/molecules26247533)
Supplement: Supplementary file 1 [file molecules-26-07533-s001.zip › molecules-1480855-supplementary.pdf]

Supplementary Material

Optimization of S-nitrosocaptopril Monohydrate Storage Conditions Based on Response Surface Method

#Lingyi Huang [1], #Yu Zhou [2,5], Yizhi Wang [3,] and *Min Lin [4,5]

Table S1. Significance analysis of Plackett-Burman experimental design results.

Factors	sum of square	Degree of freedom	Mean Square	F value	P value	The order of significance
Model	20032.38	5	4006.48	57.8300	< 0.0001	
X_1	2.52	1	2.52	0.03600	0.8550	5
X_2	372.30	1	372.30	5.3700	0.0596	3
X_3	436.81	1	436.81	6.3000	0.0458	2
X_4	19217.60	1	19217.60	277.3800	< 0.0001	1
X_5	3.14	1	3.14	0.0450	0.8384	4
Residual	415.69	6	69.28			
Sum	20448.07	11				

Table S2. Results of central combination analysis of variance.

Variance source	Sum of squares	Degrees of freedom	Mean square	F value	P value
Model	110.1516	9	12.23906	48.78216924	< 0.0001
A-Temperature	9.879013	1	9.879013	39.37553559	0.000414
B-Nitrogen purity	11.045	1	11.045	44.02290113	0.000295
C-Deoxidizer	3.577813	1	3.577813	14.26036088	0.006926
AB	0.2401	1	0.2401	0.956984931	0.360538
AC	0.081225	1	0.081225	0.323744694	0.587149
BC	1.7689	1	1.7689	7.050440001	0.032691
A^2	49.2696	1	49.2696	196.3776219	< 0.0001
B^2	15.25204	1	15.25204	60.79124001	0.000107
C^2	11.40418	1	11.40418	45.45451757	0.000267
Residual	1.756245	7	0.250892		
Lack of fit	1.234325	3	0.411442	3.15329297	0.148145
Error	0.52192	4	0.13048		
Total deviation	111.9078	16			
